# The journey of lung cancer patients from symptoms to diagnosis in Greece. A mixed methods approach

**DOI:** 10.1038/s41533-024-00359-w

**Published:** 2024-04-29

**Authors:** Ioanna Tsiligianni, Antonios Christodoulakis, Alexia Monastirioti, Dimitrios Mavroudis, Sofia Agelaki

**Affiliations:** 1 Department of Social Medicine, School of Medicine, University of Crete, Crete, Greece; 2 Department of Nursing, School of Health Sciences, Hellenic Mediterranean University, Crete, Greece; 3 Department of Medical Oncology, School of Medicine, University of Crete, Crete, Greece

## Abstract

The early diagnosis of lung cancer improves the probability of successful treatment. However, patients and physicians face several difficulties that can considerably delay the diagnostic process. A mixed-methods study that would follow the patient’s journey throughout the diagnostic process could alleviate these difficulties. This study aimed to (a) track the patients’ journey from the onset of symptoms until diagnosis and, (b) explore the patients’ perspective of the journey until diagnosis, on the largest island of Greece. A convergent mixed-methods study was conducted with 94 patients with lung cancer. Patients completed a self-report questionnaire and were interviewed about their symptoms and journey through the healthcare system before their diagnosis. Our findings revealed several problems and delays in the diagnostic process. Both quantitative and qualitative data showed that patients did not recognize their symptoms and sought medical advice in time because they overlooked or attributed their symptoms to ‘simpler’/‘more common’ causes. Furthermore, most patients were diagnosed 1–3 months after their first visit to a physician for their symptoms. Qualitative data analysis revealed three broad categories of problems that delayed diagnosis: (1) physician missteps, (2) administrative problems, and (3) the effect of the Covid-19 pandemic. This study found that major issues and delays prolong the diagnostic process for lung cancer. Therefore, optimization of diagnostic processes at each level of healthcare and interspecialty cooperation programs are needed. Furthermore, population-based interventions and patient education can help lung cancer patients be diagnosed early and improve their quality of life and disease outcomes.

## Introduction

In 2020, the World Health Organization (WHO) ranked cancer among the top four causes of death before the age of 70 years in 183 countries^1^. In 2020, the Global Cancer Observatory (Globocan) estimated 19.3 million new cases and 10 million cancer-related deaths worldwide^2^. Lung cancer is the second most common type of cancer after breast cancer and has the highest mortality rate^2–4^. High mortality results from several factors such as delays in diagnosis^5–7^, accessibility of the healthcare system, age, comorbidities, therapy complications, and the evolution of common metastatic diseases^8^. The prognosis of lung cancer can be improved by early diagnosis and treatment, which improves the quality of life, survival rates, healthcare costs, and decreases complications^9–12^.

The optimal time (for reducing the associated complications) from the first visit to a physician to the final referral to an oncologist (with diagnosis) is recommended to be a maximum of 14 days for lung cancer^13^. However, physicians strive to diagnose lung cancer quickly, because they experience numerous difficulties that delay this process^9,10,14,15^. First, patients generally relate the symptoms of lung cancer to other less serious and common medical conditions; therefore, they do not immediately seek medical attention^6^. Second, medical professionals may have misinterpreted the symptoms of lung cancer as those of other lung diseases, thereby prolonging the time to diagnosis^11^. Additionally, physicians often refer patients to different specialties for further evaluation, resulting in additional delays^9–11,16^. These difficulties are among the most common that lung cancer patients and physicians experience before diagnosis and can be improved, especially in primary care settings^5^. Furthermore, since austerity reduces population-based interventions because of a lack of funds to invest and given the increase in lung cancer cases that necessitates population-based interventions; southern European countries such as Greece should find ways to improve their healthcare facilities^17,18^.

Quantitative studies have attempted to identify difficulties and improve the detection time for lung cancer at all levels of healthcare^19–21^. However, difficulties before diagnosis persist; therefore, a different approach is needed to better understand the mechanisms that cause and/or amplify them, thus improving the detection time. A mixed-methods study that examines the journey of patients with lung cancer through the healthcare system before diagnosis could provide valuable information on how to optimize healthcare systems, educate patients and physicians, and reduce the time to diagnosis. Therefore, the present study had two objectives: (a) to examine the patient’s journey from the onset of symptoms to diagnosis and (b) to explore the patient’s perspective of the journey until diagnosis, on the largest island of Greece.

## Methods

### Study design, setting, and population

The present study used a convergent mixed-methods design. This means that both qualitative and quantitative data were collected in parallel, analyzed separately, and finally interpreted together^22^. Quantitative data were generated from a self-report questionnaire, and qualitative data were obtained through semi-structured interviews after the completion of the questionnaire. The interviews focused on the patient’s symptoms before seeking a physician’s evaluation and their journey through the healthcare system until diagnosis. Subsequently, two researchers analyzed the results separately and compared their analyses. From October 1, 2020, to September 20, 2021, 94 patients with lung cancer voluntarily participated in this study. The patients were outpatients or were treated for early, locally advanced, or metastatic lung cancer. Additionally, 43 patients declined to participate in the study [unable/overwhelmed due to end-stage disease (*N* = 11), unaware (*N* = 7) of their disease, or unwilling to participate in the study (*N* = 25)]. These were all the patients (*N* = 137) with lung cancer who were treated in the only tertiary care hospital of the study’s health region, at the aforementioned time.

### Questionnaire items and measures

A 100-item questionnaire was developed, following a thorough literature review. The questionnaire included demographic questions (gender, marital status, and age), experienced symptoms, the time between the initial experienced symptom and the first report to a physician, healthcare facilities visited/used before diagnosis, the different specialties addressed by patients before diagnosis, and the period of time from the first report to the final diagnosis. The questionnaire took ~30 min to complete and did not collect any identifiable patient data, thereby ensuring confidentiality.

### Statistical methods

Descriptive analysis of the quantitative data was performed using IBM SPSS version 28.0. Content analysis was used for the qualitative data, meaning that the data were coded, sorted, and synthesized to generate themes and categories. Subsequently, the themes and categories were analyzed by two authors by contrasting and discussing until a consensus was reached^23^.

The reliability of the study was evaluated based on four criteria: credibility, dependability, conformability, and transferability^24^. Credibility was ensured by random peer debriefing of interview transcriptions and by consensually interpreting the data. Furthermore, the interviewer was a biologist with 5 years of research experience, who had also completed a training course on qualitative methods and “how to interview cancer patients”. Dependability was ensured because the same biologist transcribed the interviews and wrote reflective notes for each interview. Conformability was ensured because data analysis and interpretation were performed independently by two authors, and then the results were discussed until they reached a consensus. To ensure transferability, the study methodology, the data collection process, and the framework were documented. It should be noted that the questionnaires and interview recordings were stored securely inside the university, and only two authors could access them. After the analyses were completed, both the qualitative and quantitative data were used for triangulation (convergence). This process creates a/n figure/illustration, where both types of data converge or diverge.

### Ethics statement

The Research Ethics Committee of the University General Hospital of Heraklion (protocol no. 394/09/13-05-2020) and the relevant ethics committee of the University of Crete (protocol no. 67/21.03.2019) approved this study. In addition, before distributing the questionnaires, an informed consent form and information on the study goals were provided and signed voluntarily by all participants. Furthermore, this study was carried out in accordance with the Declaration of Helsinki.

### Reporting summary

Further information on research design is available in the Nature Research Reporting Summary linked to this article.

## Results

### Quantitative results

The present study included 94 patients who used the services of the medical oncology department of the participating university hospital. The majority of the participants were male (*N* = 76), married (85.1%, single:11.7%, and widowed:3.2%), and had an average age of 67 years (mean age of males = 67.5, females = 62.5). The patients stated that in 12.8% (*N* = 12) of the cases, the diagnosis was an incidental finding resulting from their regular annual checkup or in the context of preoperative evaluation for surgical procedures. However, 87.2% (*N* = 82) stated that (before investigation and diagnosis), they experienced symptoms related (75.5%, *N* = 71) or unrelated (11.7%, *N* = 11) to their disease.

### Symptoms before seeking medical evaluation

**Fig. 1:**
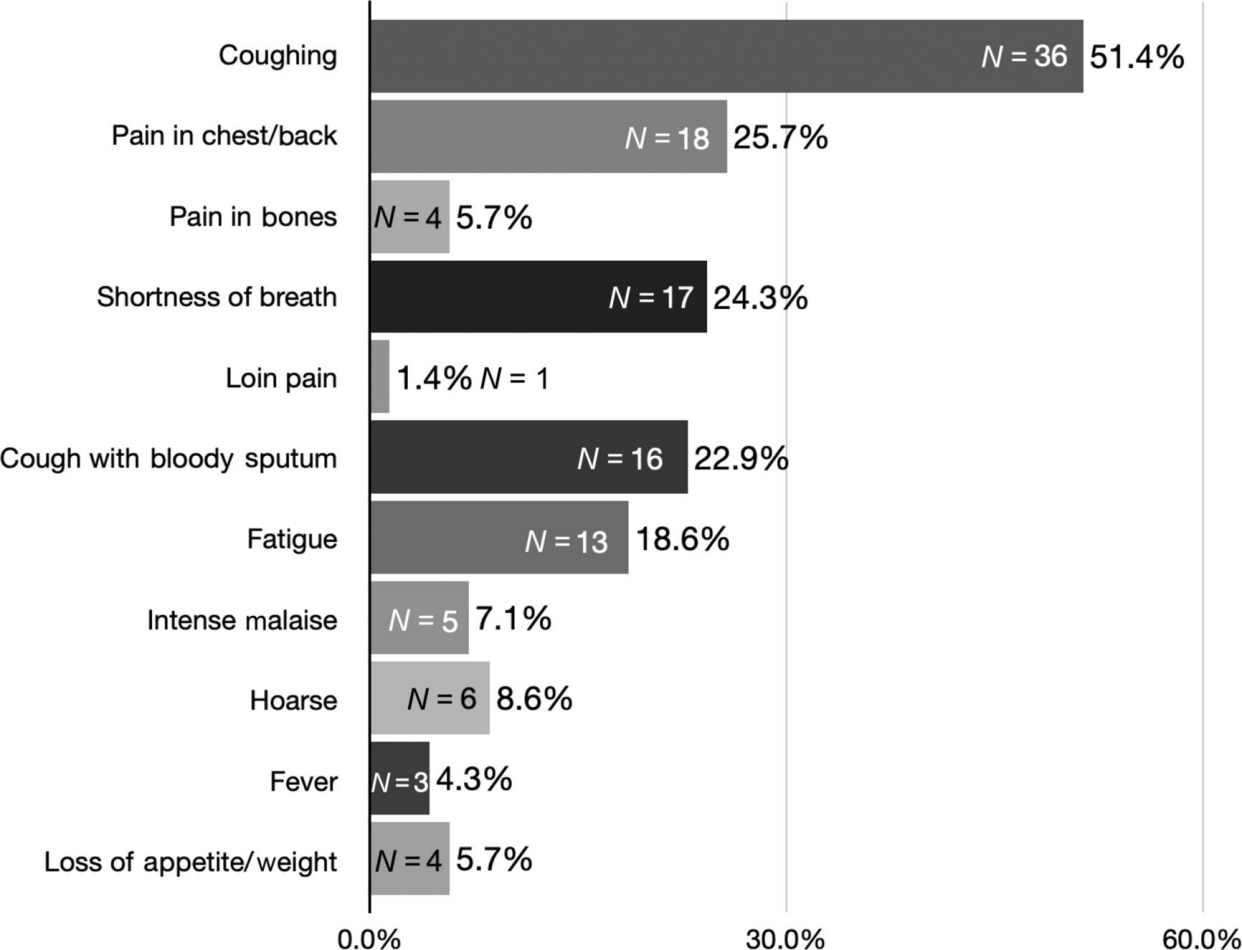
The symptoms* patients experienced before they sought a physician and were related to lung cancer (*Ν* = 71). *Each symptom reported by the patients is presented as binary (yes, I experienced it or no, I did not experience it).

The patients (*N* = 82) experienced different symptoms before seeking an evaluation from a physician. Among the patients who experienced symptoms related to lung cancer (*N* = 71), the most common was cough (51.4%). Additionally, these patients (*N* = 71) reported having experienced multiple symptoms before investigation and diagnosis: pain in the chest/back (25.7%), shortness of breath (24.3%), cough with bloody sputum (22.9%), fatigue (18.6%), hoarseness (8.6%), intense malaise (7.1%), bone pain (5.7%), loss of appetite/weight (5.7%), fever (4.3%), and loin pain (1.4%) (Fig. 1).

### Time from symptoms to physician visit

**Fig. 2:**
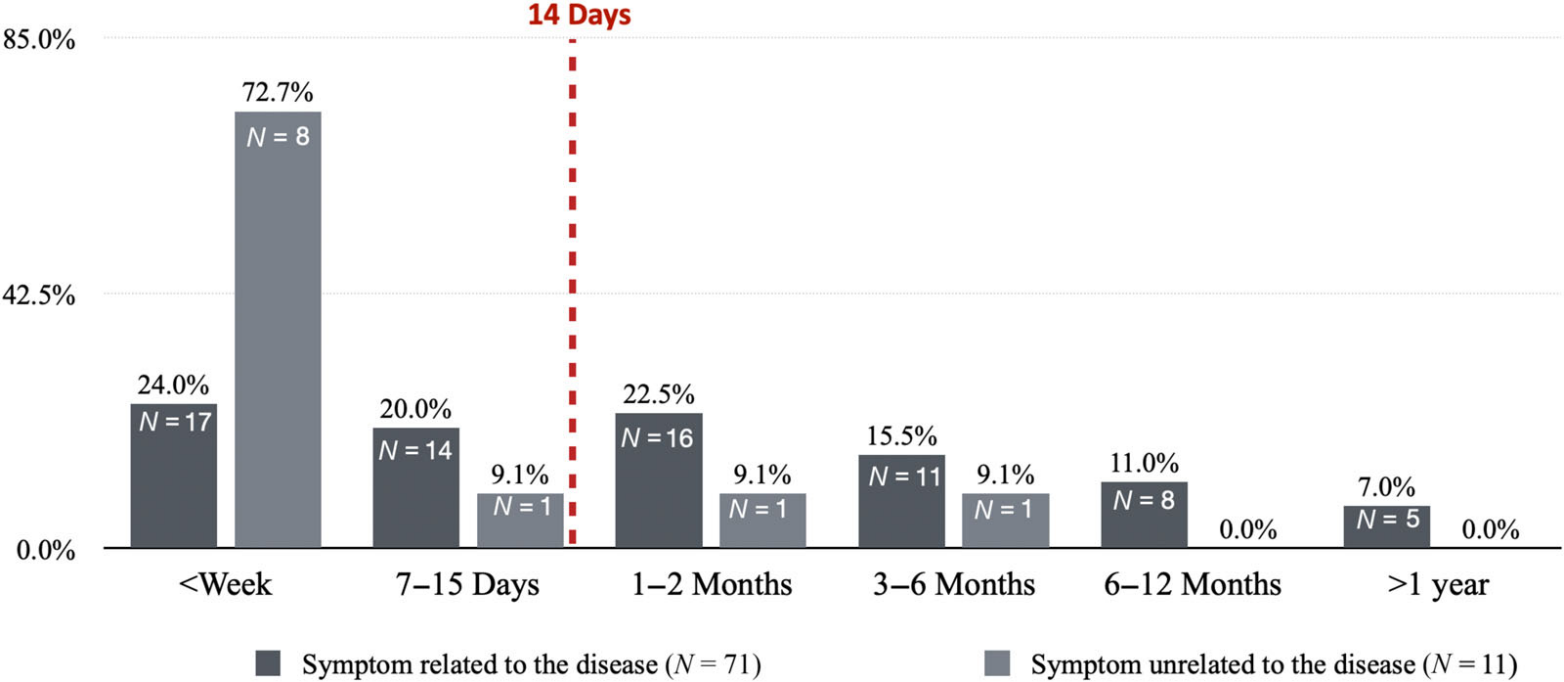
Time between the onset of a symptom/s and the first report of it/them to a physician.

The time between the onset of the symptom(s) (first presentation of symptom/s) and the first report to a physician (recorded relevant consultation) varied considerably for symptoms related and unrelated to the disease. Patients reported disease-unrelated symptom(s) earlier than related symptoms to physicians (<week:76.9% vs. 24.3%, 7–15 days:7.7% vs. 18.6%, 1–2 months: 7.7% vs. 22.9%, 3–6 months:7.7% vs. 15.7%, 6–12 months 0% vs. 11.4%, and >1 year: 0% vs. 7.1%) (Fig. 2).

### The facilities patients used during their journey

**Fig. 3:**
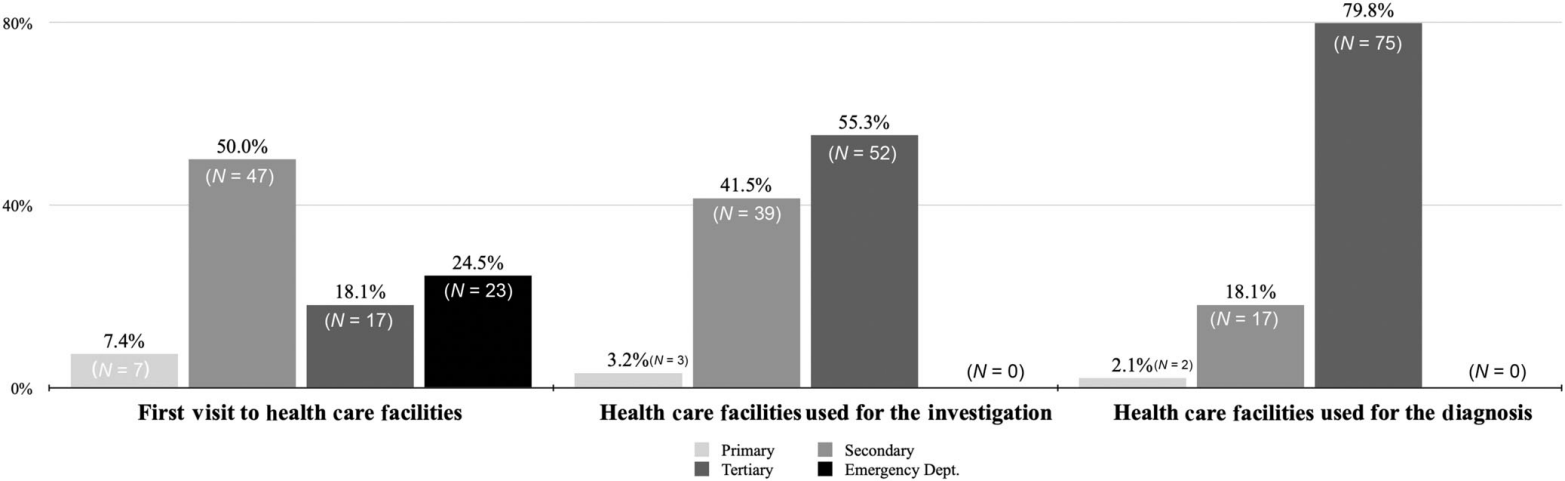
Health care facility used for first visit, investigation, and diagnosis of lung cancer.

Patients visited different healthcare facilities from disease onset to diagnosis. During their first visit, most patients used a secondary healthcare facility (50%), while the rest chose an emergency department (24.5%), a tertiary healthcare facility (18.1%), or a primary healthcare facility (7.4%) (Fig. 3). For medical examinations, the majority of patients used a tertiary health care facility (55.3%) or a secondary health care facility (41.5%), although few patients used a primary health care facility (3.2%). For the diagnosis, most patients used a tertiary healthcare facility (79.8%), while some patients visited a secondary healthcare facility (18.1%) and others visited a primary healthcare facility (2.1%) (Fig. 3).

### Specialties visited before the oncologist

**Fig. 4:**
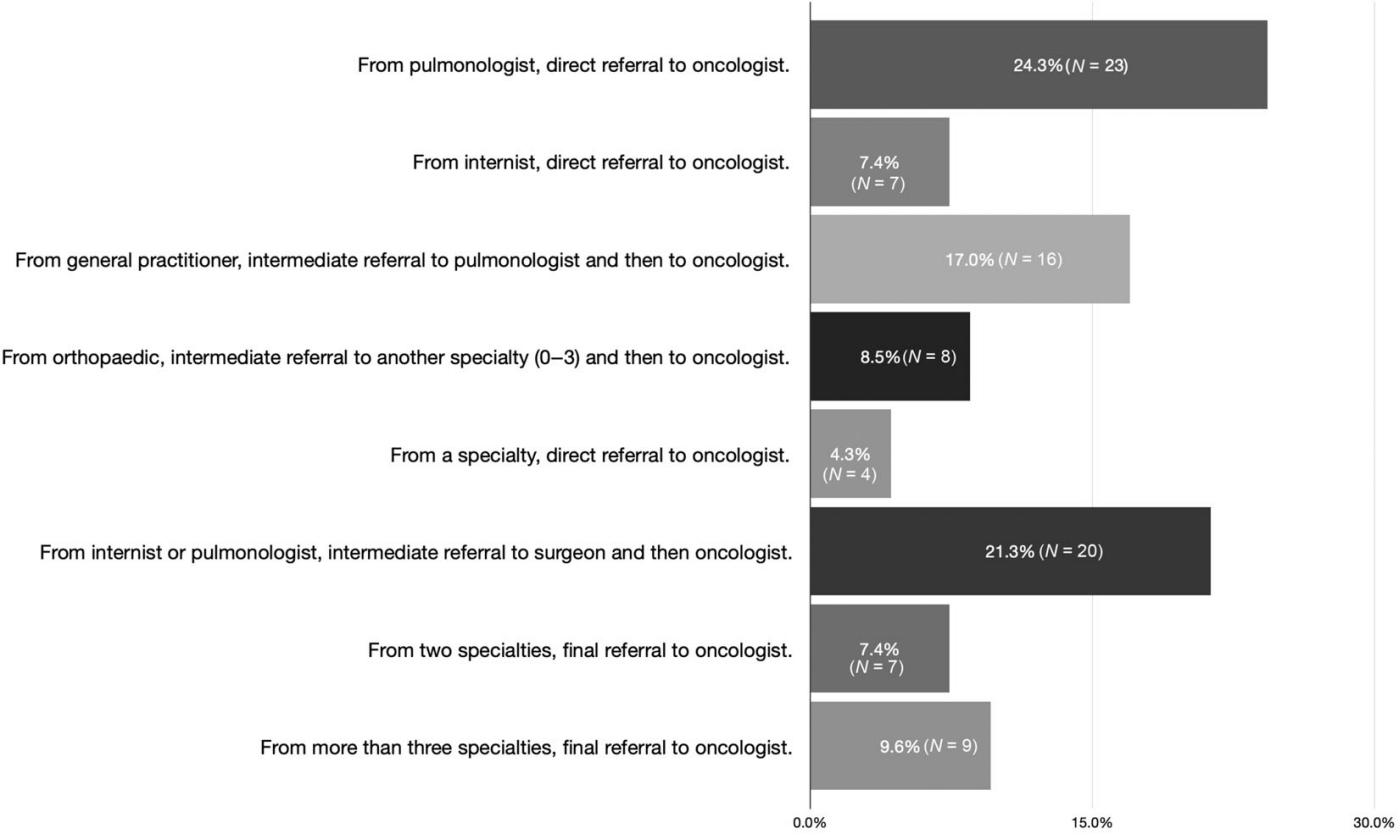
The different specialties to which patients were referred as their first visit to a physician, and the number of intermediate specialties up to the oncologist.

The patients were referred to different specialties from their first visit to a physician for evaluation by an oncologist. Most patients were referred from one specialty to an oncologist, with an intermediate specialty between (45.7%) and some patients were directly referred from one specialty to an oncologist (36%) (Fig. 4). However, 8.5% were referred by an orthopedic surgeon to an oncologist with 0–3 intermediate referrals to other specialties, and 9.6% were referred to an oncologist from more than three different specialties (Fig. 4).

### Time from first visit to diagnosis

**Fig. 5:**
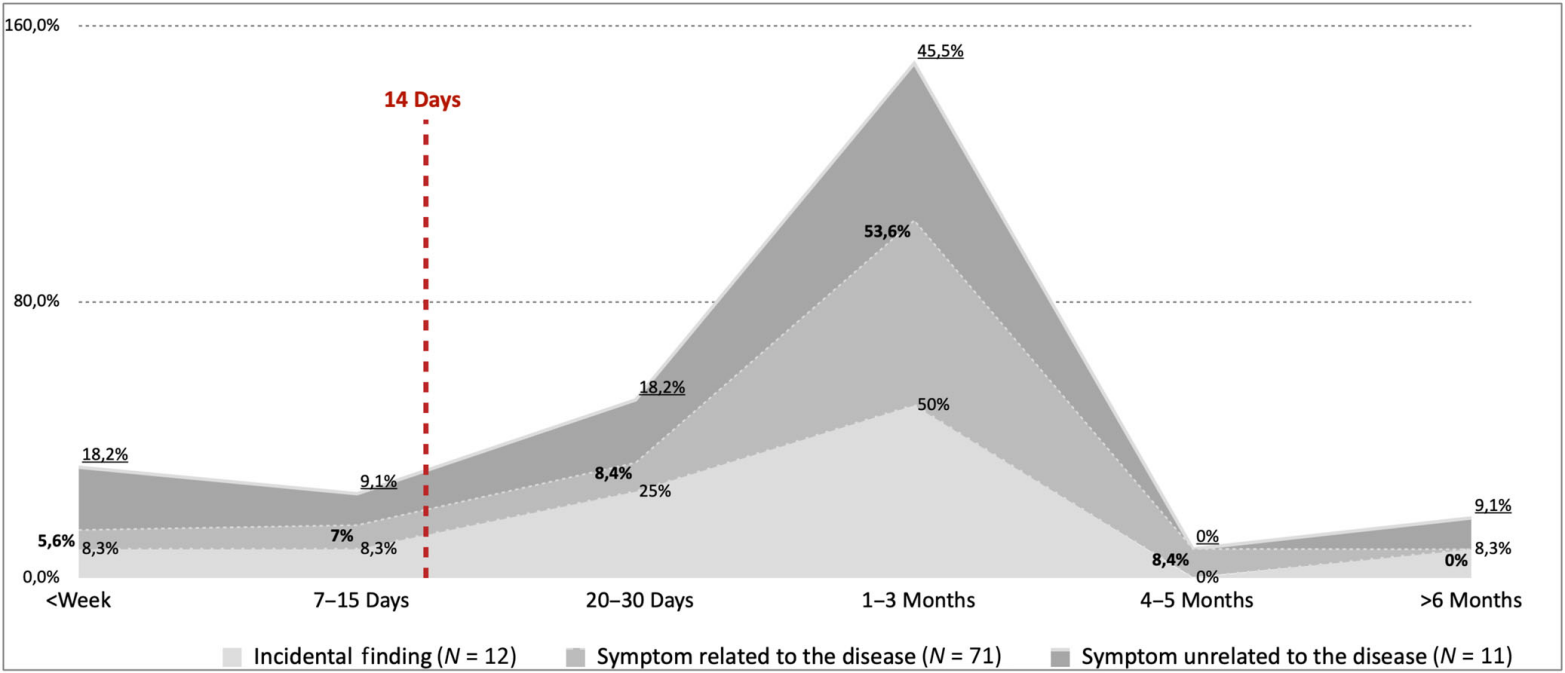
The time between the first visit to a physician, either for a scheduled check-up or due to symptoms, until the final diagnosis.

The time between the first visit to a physician (for a scheduled check-up or symptoms) and the final diagnosis varied by patient. Patients (*N* = 12) whose lung cancer was diagnosed as an incidental finding visited a physician once before diagnosis. The time from first visit to diagnosis for the remaining patients (*N* = 82) was 1–3 months (symptoms unrelated to the disease 46.2%, symptoms related to the disease 52.9%, and incidental finding 45.5%), and for many patients the time was 20–30 days (symptom unrelated to the disease, 23.1%; symptom related to the disease, 25.7%; and incidental finding, 27.3%) (Fig. 5). In addition, several patients had their final diagnosis in less than a week (symptoms unrelated to the disease 15.4%, symptoms related to the disease 5.7%, and incidental finding 9.1%), or 7–15 days (symptom unrelated to the disease 7.7%, symptoms related to the disease 7.1%, and incidental finding 9.1%). However, some patients did not achieve a diagnosis for 4–5 months (symptoms related to the disease 8.6%) with three (3) patients not achieving a diagnosis for more than 6 months (symptoms unrelated to the disease, 7.7%; incidental finding, 9.1%) (Fig. 5).

### Qualitative phase

After completing the questionnaires, all 94 quantitative phase participants participated in semi-structured interviews. The interviews revealed two themes: the symptoms that patients endured before seeking medical attention and the journey they experienced through the healthcare system before diagnosis (Table 1).

**Table 1:** Qualitative themes, categories, and example quotes.

Theme	Category	Example quote
The symptoms before seeking medical attention	Respiratory symptoms	“Before the diagnosis, I had a persistent cough for some time. I thought it was due to COPD so I started taking antibiotics, antitussives, and inhalers on my own. Eventually, the hemoptysis started. Then I visited the doctor.”
		“I had chronic shortness of breath. But some nights I couldn’t breathe.”
		“I felt like I had something stuck in my throat, I was trying to cough and “break” it. If I had gone to the doctor, then I could have been saved. It was a premonition.”
		“I coughed for almost 2 years. I used to smoke and thought that’s why I coughed. Towards the end, I also had back pain. At first, I thought it was because of work. Eventually, I went to the ER.”
		“I thought it was due to smoking. I wasn’t paying attention. It didn’t affect me in any way.”
		“I had to cough up blood to go see the doctor.”
	Pain symptoms	“My cousin also had back pains from work. I thought it was the same for me. It lasted 15 days. Then I went to the hospital.”
		“After I was diagnosed, I remembered feeling a “pinch” in my chest on the side where I have the tumor like it was a nerve pain.”
	Psychological symptoms	“My father didn’t eat. He was not in a good mood. This went on for three months. But he wouldn’t go to the doctor. He only went to get the flu shot.”
		“Dad doesn’t know that he has cancer. Three months before the diagnosis, he was very depressed.”
The journey within the healthcare system	Physicians’—healthcare system missteps	“I had symptoms, but I didn’t pay attention. I went to the hospital with an allergy. They treated me and I got burns on my hands.”
		“When my sister got sick, I went to get a checkup as well. Of course, the pulmonologist who saw me only did an x-ray and spirometry, and nothing showed up. But I don’t think he looked at them properly.”
		“The pulmonologist I first visited dismissed me before he’d seen the tests. He said to come back after three months. Of course, I found another doctor.”
		“I regularly visit a GP in the health center. The tests I did, have shown cancer for years. He never told me.”
		“If it wasn’t for that unacceptable doctor at another Hospital, I would have found the problem earlier. However, the local primary health care center I visited the next time for my symptoms was excellent.”
		“From the very beginning when I started with the tests, I visited a surgeon. If I hadn’t been presented with the thyroid problem, I wouldn’t have known that I needed to visit an oncologist.”
		“I was feeling some dizziness before I was diagnosed, I thought it was my eyesight. The ophthalmologist, of course, had attributed it to age at the time.”
		“The doctors killed me. They gave me the wrong drugs for months. I had a blood clot in my leg, they finally cut it off at the knee. I don’t care about the cancer. It’s my leg that makes me sad.”
		“Suddenly, I got a hoarse voice. I went to a general practitioner to see me. He gave me pills and spray. But my voice didn’t come back.”
		“I went to a bunch of doctors only in the end to be told that I have cancer. They even sent me to a plastic surgeon.”
		“For 1.5 years after the surgery, I visited a surgeon. I didn’t know I was supposed to visit an oncologist. No one told me. At some point, I started having instability. Now I visit an oncologist because I have metastases in my head.”
	Administrative problems	“They gave me a hard time at the hospital until they could get me an appointment to have my tests.”
		“The biopsy took too long to come out.”
		“I was sent to have a biopsy at a private center. They asked for 1500€. At the hospital, it was almost impossible to get an appointment for a bronchoscopy.”
		“CT scan should be included in the primary care.”
	The effect of Covid-19 pandemic	“I couldn’t make an appointment for tests so that I could start my treatment because of the lockdown.”
		“I had finished treatments. In February 2020 I had a re-examination. A change in the lung was found. I had to have additional tests. Due to Covid-19 they wouldn’t let me make an appointment at the hospital. In June when we were now allowed to go, I returned to the hospital with metastases in my head.”
		“Because of the covid, they kept canceling my test appointments. It took me 3 months to start treatment.”

### Theme 1: The symptoms before seeking medical attention

The interviews revealed that patients with lung cancer experienced several symptoms prior to visiting a physician. Thematic analysis revealed three categories of these symptoms: respiratory, pain, and psychological (Table 1). Respiratory symptoms were the most prevalent in our interviews, especially for different types of cough, which were usually overlooked until the first incidence of hemoptysis (Table 1). Pain was usually attributed to work/exercise or some type of neuropathy by patients. Interestingly, the patients reported experiencing several types of psychological symptoms, especially depressive symptoms, a few months before diagnosis (Table 1).

### Theme 2: The journey within the healthcare system

During the interviews, the patients were asked to describe their journeys within the healthcare system. Our analysis revealed three broad categories that described their journey: physician missteps, administrative problems, and the effect of the Covid-19 pandemic (Table 1). First, physician missteps were the most common, with misdiagnosis, mistreatment, wrong referrals, apathy, and impoliteness (inappropriate behavior) being more prevalent. Second, patients also reported administrative problems such as the inability to make an appointment with their physicians, delays in test results, and outpatient referrals to other centers for further testing. Third, they reported that the Covid-19 pandemic affected the diagnostic process because patients were unable to book appointments and complete their tests or re-examinations, which delayed their treatment (Table 1).

### Triangulation analysis

**Fig. 6:**
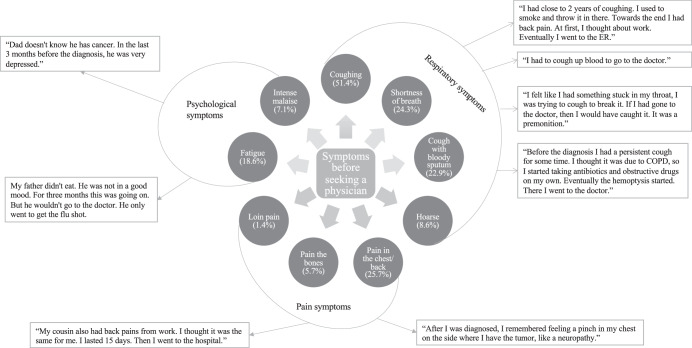
The symptoms before seeking a physician.

Finally, the results of the interviews converged with the quantitative data, resulting in a multifaceted illustration of the journey that patients with lung cancer experienced from the onset (Fig. 5) of disease symptoms until diagnosis (Fig. 6). It should be noted that in Fig. 5, we excluded the symptoms of ‘fever’ and ‘loss of appetite/weight’ since they diverged from the results of the qualitative data (categories).

## Discussion

The present study aimed to (a) examine the patient’s journey from the onset of symptoms to diagnosis and (b) explore the patients’ perspective of the journey until diagnosis, on the island of Crete in Greece. Our findings revealed major delays in the patient’s journey from the onset of symptoms to diagnosis because patients overlooked symptoms. Furthermore, through interviews and quantitative data, the major problems (physician missteps, administrative problems, and the effect of the Covid-19 pandemic) were revealed and elaborated upon by the patients, which delayed the diagnostic process. Interestingly, the present study was conducted in Crete, where primary healthcare facilities are well-developed (approximately 250–300 GPs for 630,000 people). However, only a handful of patients chose primary care facilities for their first visit, examination, and diagnosis of lung cancer (Fig. 3). It is important to highlight that Greece does not have an official lung cancer pathway. However, the ideal pathway for lung cancer patients begins by visiting primary care for diagnosis. From there, patients are referred to an oncologist who will evaluate their condition, develop a treatment plan, and provide necessary supportive care. The oncologist will also ensure proper follow-up for the patient.

A major finding of the present study was that respiratory symptoms (particularly coughing) and pain symptoms were the most common (Fig. 1) before the patient’s initial report to a physician. This finding is supported by other studies that examined the initial symptoms experienced by patients with lung cancer before seeking medical evaluation^19,20,25–27^. Primary care physicians should be vigilant, as pain is often overlooked when diagnosing lung cancer. Furthermore, our qualitative results revealed that the patients underestimated their initial symptoms until their cancer had already progressed to a more advanced stage. Remarkably, patient #34 said “Before the diagnosis I had a persistent cough for some time. I thought it was due to COPD, so I started taking antibiotics, antitussives, and inhalers on my own. Eventually, hemoptysis started. At that point, I visited the doctor”. Studies have shown that patients have fragmented knowledge about lung cancer symptoms and usually attribute them to other factors^28–30^, which delay them from seeking medical attention. In addition, patients could have appraised their symptoms based on previous experiences and/or knowledge and sought medical attention when they could no longer explain their symptoms^31^. Another interesting explanation could be that former or passive smokers underestimated the risk of lung cancer, which delayed them from seeking medical attention^32^. This explanation is also reinforced by patient #1, who said: “I had close to two years of coughing. I used to smoke and thought that’s why I coughed, towards the end I also had back pain. At first, I thought it was because of work. Eventually, I had to visit the Emergency Department”. These explanations underline the need for primary healthcare providers to better inform their patients about the risk factors for lung cancer and the value of prompt evaluation^33^.

Another major finding of our study was the illustration of a lung cancer patient’s journey from the first visit to a physician until diagnosis. We found several problems that delayed the process, such as multiple referrals, diagnostic missteps, administrative problems, and delays owing to the Covid-19 pandemic. Interestingly, a study has shown that Greece’s primary healthcare practitioners investigate lung cancer more often than other Balkan countries^34^. However, lung cancer is difficult to diagnose because it can have an atypical presentation^30^ and even a normal chest x-ray^35^, which can explain the multiple referrals. Another possible explanation for multiple referrals and diagnostic missteps could be the underuse of low-dose computed tomography in high-risk individuals for lung cancer screening by primary care physicians^15,36^. Nevertheless, a testimonial from patient #2: “I went to a bunch of doctors only in the end to be told that I have cancer. They even sent me to a plastic surgeon” illustrates the extent of the problem. Unfortunately, we could not find any study to explain the administrative problems; this means that more studies are required to further investigate such problems and propose solutions. However, it is worth mentioning that Greece has a national health system, there is also private healthcare, that is paid either with private contracts with the patients or directly with money out of pocket. Therefore, without a referral from a primary care physician can either pay specialists privately or they can visit the national health system without paying. However, it is known that the Greek national health system has faced many difficulties due to many years of austerity^37^. The Covid-19 pandemic probably amplified diagnostic missteps and administrative problems, as patient #32 reported: “Because of the covid, they kept canceling my test appointments. It took me 3 months to start treatment”. Moreover, studies confirm our findings, since major delays in the diagnostic process of multiple types of cancer were associated with the pandemic, such as colorectal, breast, and lung cancers^38–41^. Finally, these findings contradict the proposed optimal path for lung cancer patients through the healthcare system^5^. More specifically, the level of awareness in the patients of this study was low maybe because there was a lack of education in recognizing their symptoms early and seeking medical attention when the disease had irreversibly progressed. Additionally, healthcare professionals further delayed the diagnostic process through misdiagnosis, multiple referrals, and so on. These delays in our sample resulted in an average time of 1–3 months for the diagnosis of lung cancer (Fig. 5), which is much longer than the recommended 14 days (optimal time)^13^.

The findings of the present study suggest that a major change in the Greek and in similar healthcare systems is urgently needed to drastically reduce the time from the first visit to diagnosis, especially in primary care. Therefore, we propose a two-step solution to reduce the time required for diagnosis. First, healthcare authorities should educate healthcare professionals at all levels to recognize the symptoms of lung cancer^42^. Second, healthcare authorities should educate primary healthcare professionals to better inform patients/community members of the symptoms and risk factors associated with lung cancer. Toward this end, we produced two guidance booklets for healthcare providers and the general population, which were distributed by regional health authorities. Third, a Lung cancer pathway like the UK National Optimal Lung Cancer Pathway (NOLCP)^43^ and the NICE Faster Diagnosis Framework^44^ so that Greece could incorporate a national approach. Fourth, there is a pressing need to conduct implementational studies in Greece for lung cancer screening, in accordance with established guidelines. Such studies have the potential to significantly decrease the time taken for diagnosis and may also serve as a catalyst for political decisions regarding nationwide screening programs, which are currently unavailable in Greece^45^. Finally, there is a need for more research to help overcome the barriers to implementing low-dose computed tomography for lung cancer screening, such as false-positive tests, overdiagnosis, and the negative psychological impact of screening^45^.

To the best of our knowledge, this is the first study to examine and portray the journey of lung cancer patients from the onset of symptoms until diagnosis using a mixed methods study design. However, multiple problems were found, emphasizing the need to immediately redesign primary healthcare lung cancer diagnostic protocols. Additionally, our study had a few limitations inherent to the mixed-methods design. Recall bias may have affected the quantitative data, especially for the first symptoms, owing to an unknown time since diagnosis. For the qualitative data, we may have fallen into participant and/or researcher bias(es). Although, as explained in the “Methods” section, an educated and experienced interviewer reassured patients of their answers’ confidentiality to mitigate those bias(es). Finally, the present study was single-center and did not follow up on patients to ascertain how these delays affected their disease outcomes. To this end, multicenter longitudinal studies could better assess the outcomes of these delays.

In conclusion, the present study depicts the journey of patients with lung cancer from the onset of the disease to diagnosis through the healthcare system. Our findings clearly indicate areas that can be improved to reduce the time to diagnosis. Healthcare professionals and managers should utilize this knowledge to reexamine and optimize the way in which each level of healthcare operates. Additionally, physicians can better inform their patients and improve cooperation among specialties. In doing so, physicians should be able to diagnose lung cancer more quickly and improve the quality of life of their patients and the outcomes of the disease.

### Supplementary information

Reporting Summary

## Data Availability

The data presented in this study are available upon request from the corresponding author.
